# Inflammatory Markers in Women with Endometriosis Before and After the COVID-19 Pandemic: A Matched Cohort Study from the Tehran Lipid and Glucose Study

**DOI:** 10.5812/ijem-171933

**Published:** 2026-06-23

**Authors:** Maryam Matouri, Ameneh Koochaki, Shahla Noori Ardebili, Mostafa Haji Molla Hoseini, Marzieh Saei Ghare Naz, Moein Malekzadeh, Maryam Mousavi, Fahimeh Ramezani Tehrani, Nariman Mosaffa

**Affiliations:** 1 Department of Immunology, School of Medicine, Shahid Beheshti University of Medical Sciences, Tehran, Iran; 2 Reproductive Endocrinology Research Center, Research Institute for Endocrine Molecular Biology, Research Institute for Endocrine Sciences, Shahid Beheshti University of Medical Sciences, Tehran, Iran; 3 Department of Immunology, Golestan University of Medical Sciences, Gorgan, Iran; 4 Foundation for Research and Education Excellence, Vestavia Hills, Al, USA

**Keywords:** Endometriosis, COVID-19, Inflammation

## Abstract

**Background:**

Endometriosis (EM) is a common chronic inflammatory disorder affecting women. The coronavirus disease 2019 (COVID-19) pandemic may have been associated with changes in systemic inflammatory regulation.

**Objectives:**

This study aimed to measure and compare serum interleukin-6 (IL-6) levels, mRNA levels of vascular endothelial growth factor (VEGF), C-X-C motif chemokine 5 (CXCL5), and substance P (SP), as well as markers of neutrophil extracellular trap formation (NETosis), in patients with EM before and after the onset of the COVID-19 pandemic.

**Methods:**

We used data from the Tehran Lipid and Glucose Study (TLGS). Among 2558 women in the TLGS, 465 had a diagnosis of endometriosis. Of these, 13 women had biobanked samples available from both the pre-pandemic (Phase 6, 2016 - 2018) and post-pandemic (Phase 7, 2021 - 2023) periods and were included as matched pairs within the same individuals. The primary exposure was the post-pandemic calendar period; individual COVID-19 infection data were not collected. Real-time PCR was used to quantify the expression of genes associated with SP (TAC1), neurokinin 1 receptor (TACR1), VEGF, and CXCL5 in peripheral blood mononuclear cells. Markers of NETosis, including neutrophil elastase (NE), myeloperoxidase (MPO), peptidyl arginine deiminase 4 (PAD4), and matrix metallopeptidase 9 (MMP9), were assessed. Serum IL-6 concentrations were measured by enzyme-linked immunosorbent assay.

**Results:**

Gene expression analysis showed no significant changes in TAC1, TACR1, VEGF, or CXCL5; all 95% confidence intervals (CIs) included unity, and the study was underpowered to detect small differences. Evaluation of NETosis-related genes in neutrophils stimulated with patient sera showed no significant differences in PAD4, MMP9, or MPO expression; however, NE expression was significantly elevated after the pandemic (fold change = 1.5; 95% CI, 1.01 to 2.25; P = 0.048). Other NETosis markers and IL-6 did not differ significantly (eg, IL-6 median paired difference, +0.58 pg/mL; 95% CI, -0.89 to +2.34 pg/mL; P = 0.750); thus, all non-significant results are inconclusive.

**Conclusions:**

A preliminary signal of increased neutrophil elastase expression was observed after the pandemic, whereas other inflammatory and NETosis markers remained inconclusive. In this small investigation, the relative contributions of COVID-19 infection, vaccination, psychosocial stress, and lifestyle changes could not be disentangled. These hypothesis-generating findings require confirmation in larger, well-characterized cohorts.

## 1. Background

Endometriosis (EM) is an inflammatory disorder with an estimated prevalence of 10%–15% among women of reproductive age and is characterized by the ectopic growth of endometrial-like tissue (1). EM causes debilitating symptoms, including chronic pelvic pain, dysmenorrhea, and infertility (2). Its pathogenesis involves complex interactions among immune dysfunction, angiogenesis, and neurogenic processes. Although retrograde menstruation is the primary mechanism for the dispersal of endometrial cells, successful ectopic implantation requires additional factors, including angiogenesis, lymphangiogenesis, and neurogenesis (3).

Interleukin-6 (IL-6) enhances angiogenesis and pain signaling, whereas interleukin-1 beta (IL-1β) stimulates the production of inflammatory factors involved in neuroangiogenesis (4). Tumor necrosis factor alpha (TNF-α) and IL-6 increase the secretion of vascular endothelial growth factor (VEGF) from immune cells, thereby enhancing angiogenesis, which is essential for lesion survival. Chemokines also contribute substantially to disease pathogenesis by inducing pain and recruiting neutrophils to inflammatory sites during the early stages of lesion formation (4).

Neuropeptides are crucial in the pathophysiology of EM, particularly in pain mechanisms and disease progression. Calcitonin gene-related peptide (CGRP) and substance P (SP) are key mediators that, via their receptors, neurokinin 1 receptor (NK1R), calcitonin receptor-like receptor (CRLR), and receptor activity-modifying protein 1 (RAMP-1), accelerate the development and fibrogenesis of EM by inducing epithelial–mesenchymal transition and promoting fibroblast differentiation into myofibroblasts (5). Recently, Velho et al. reported that endometriotic lesions show increased innervation, with higher nerve fiber density and elevated SP expression compared with control tissues (6).

Recent research has also identified neutrophil extracellular traps (NETs), structures formed when neutrophils release decondensed chromatin and granular proteins to trap pathogens, as key molecular mediators in the pathogenesis of EM (7). Angiogenesis is enhanced by these inflammatory mediators, which also interact with sensory neurons to trigger pain signaling, suggesting that they may represent potential therapeutic targets (4, 8).

The COVID-19 pandemic has shown that COVID-19 infection can trigger severe inflammatory responses beyond the acute illness. This virus induces a cytokine storm characterized by dysregulated immune activation and excessive production of pro-inflammatory cytokines, particularly IL-6, leading to acute respiratory distress syndrome and multiorgan damage (9). COVID-19 infection also promotes excessive NET formation through NETosis, a major mechanism contributing to COVID-19 disease progression and subsequent chronic complications (10). Increased NET formation is linked to worse clinical outcomes, coagulopathy, and immunothrombosis in patients with COVID-19 (11).

COVID-19 infection may exacerbate existing EM symptoms. Recent studies have reported worsening EM symptoms after COVID-19 infection (12, 13). Evidence suggests that COVID-19 can aggravate EM symptoms, including persistent pelvic pain, menstrual pain, dyspareunia, gastrointestinal complaints, profound fatigue, and increased stress, anxiety, and depression (12).

## 2. Objectives

Given the overlapping inflammatory pathways involved in COVID-19 and EM, we hypothesized that complications of the COVID-19 pandemic, whether due to direct viral infection, increased psychosocial stress, or environmental changes, could exacerbate the inflammatory milieu in individuals with underlying EM. This study compared levels of key inflammatory factors, including IL-6, VEGF, C-X-C motif chemokine 5 (CXCL5), SP, and NETosis markers, in patients with EM before and after the COVID-19 pandemic.

## 3. Methods

### 3.1. Study Design and Sample Collection

We conducted a secondary analysis using data from the prospective TLGS cohort to compare inflammatory markers in women with EM before and after the onset of the COVID-19 pandemic. The TLGS includes multiple follow-up phases. Phase 6, conducted during 2016 - 2018, represented the pre-pandemic period, and Phase 7, with samples collected after March 2020 and specifically during 2021 - 2023, represented the post-pandemic period.

Among 2558 women in the TLGS, 465 had a diagnosis of endometriosis. Of these, only 13 women had biobanked samples available from both Phase 6 and Phase 7 and were included as 13 matched pairs from the same individuals. No additional eligible women with matched samples were excluded because of missing laboratory data; therefore, all available eligible pairs were analyzed. Each participant served as her own control, constituting a paired repeated-measures design.

The primary exposure was the post-pandemic calendar period (Phase 7, 2021 - 2023), which encompassed potential direct viral exposure, vaccination, pandemic-related psychosocial stress, and associated lifestyle changes. Individual COVID-19 infection status was not an inclusion criterion or an exposure variable because such data were not collected for this subcohort. Detailed information on COVID-19 infection history, vaccination status, COVID-19 severity, long-COVID symptoms, or quantified pandemic-related stress exposure was not available. Consequently, the exposure reflects only the post-pandemic time period and cannot be attributed solely to infection with the virus.

The diagnosis of EM was based on clinical symptoms and transvaginal or abdominal ultrasound findings, including the presence of endometriomas and/or deep infiltrating endometriosis, with surgical confirmation where available. Severity was classified according to the revised American Society for Reproductive Medicine (rASRM) staging system (stages I - IV); however, complete individual stage data could not be retrieved for all participants because surgical records were not uniformly available. Information on menstrual-cycle phase at blood sampling, hormonal therapy, anti-inflammatory or analgesic use, prior EM-related surgery, and other treatments was not systematically collected for this subcohort and was therefore unavailable. Beyond age, body mass index (BMI), and education (Table 1), no additional endometriosis-specific clinical variables, such as infertility status or comorbid inflammatory conditions, were available.

**Table 1.** Characteristics of Women Participating in the Study (N = 13 Matched Pairs) ^a^

Variables	Pre-COVID-19 Pandemic (Phase 6)	Post-COVID-19 Pandemic (Phase 7)
**Age, y**	43.42 ± 9.80	46.73 ± 10.45
**BMI, kg/m^2^**	27.66 ± 5.94	27.67 ± 6.26
**Education**		
Illiterate	8 (61.54)	6 (46.15)
Less than a high school diploma or diploma	1 (7.69)	1 (7.69)
Above diploma	4 (30.77)	6 (46.15)

^a^ Values are expressed as mean ± SD or No. (%). The mean age increased by approximately 3.3 years between phases, whereas BMI remained essentially unchanged (mean difference, +0.01 kg/m^2^). Because each participant served as her own control, formal paired significance tests for these variables were not performed.

Among potential confounders, age, BMI, and education level were available from the TLGS database. Data on EM treatment, hormonal medications, surgical history, menstrual phase, comorbidities, vaccination status, COVID-19 infection history, and potential laboratory batch effects were not available for this subcohort.

### 3.2. Evaluation of SP, NK1R, VEGF, and CXCL5 Expression Levels

Peripheral blood mononuclear cells (PBMCs) were used to quantify the expression of the target genes. The gene encoding SP is tachykinin precursor 1 (TAC1), and tachykinin receptor 1 (TACR1) encodes NK1R.

### 3.3. RNA Extraction and Quantitative Polymerase Chain Reaction

Total RNA was isolated by phenol-chloroform extraction, quantified by NanoDrop spectrophotometry, and reverse-transcribed into complementary DNA (cDNA) using a commercial cDNA synthesis kit (Yekta Tajhiz Azma, Iran), according to the manufacturer's protocol. Quantitative PCR with SYBR Green was performed on an ABI StepOne Plus Real-Time PCR System (Thermo Fisher Scientific, USA) under the following cycling conditions: 95°C for 10 minutes, followed by 40 cycles of 95°C for 20 seconds, 60°C for 30 seconds, and 72°C for 30 seconds. *Relative expression* was normalized against glyceraldehyde-3-phosphate dehydrogenase (GAPDH). All reactions were performed in duplicate, and relative expression was calculated using the 2^-ΔΔCt^ method.

### 3.4. Induction of NETosis

#### 3.4.1. Isolation of Human Neutrophils

For this study, multiple samples were collected from a single eligible donor. Neutrophils were isolated using a 2-step dextran-Ficoll gradient centrifugation. Giemsa staining was used to assess the purity of isolated neutrophils by examining nuclear morphology under a light microscope. The purity of the isolated cells was greater than 95%. Neutrophil viability was assessed by trypan blue exclusion and exceeded 90%.

#### 3.4.2. Stimulation of Neutrophils

Neutrophils were plated at 2 × 10^6^ cells/mL in a 24-well plate and incubated for 1 hour at 37°C under 5% CO_2_ to allow adhesion. Then, 400 µL of RPMI culture medium containing 10% fetal bovine serum (FBS) was added to each well. Next, 100 µL of patient serum was gently added to each well to avoid disrupting cell adhesion. A positive control was included in which neutrophils were activated by 100 nM phorbol myristate acetate (PMA), a known NETosis inducer (14), and a negative control received RPMI alone. Plates were incubated at 37°C under 5% CO_2_ for 2 hours to induce NETosis.

#### 3.4.3. NETosis Formation Assay

To investigate the induction of genes involved in NETosis, the expression of 4 key NETosis-related genes, peptidyl arginine deiminase 4 (PAD4), matrix metallopeptidase 9 (MMP9), neutrophil elastase (NE), and myeloperoxidase (MPO), was assessed using real-time PCR (Table 2), as described for PBMC samples.

**Table 2.** Primer Sequences Used for the Real-time PCR Assay ^a^

Genes	Forward	Reverse
**VEGFA**	CCCATGGCAGAAGGAGGAG	GATGGCTTGAAGATGTACTCG
**TACR1**	CCACATCTGTGTGACTGTGC	TCATCATTTTGACCACCTTGCG
**TAC1**	GACCAGATCAAGGAGGAACTGC	CATGTCCAGCATCCCGTTTG
**CXCL5**	TGTGCAATTAACAAAGCTACTGC	AGGCATCTAAAAAGCTCAGCA
**GAPDH**	CCACTCCTCCACCTTTGACG	CCACCACCCTGTTGCTGTAG
**PAD4**	CCATCCTGCTGGTGAACTGT	GTCCTTGGGGGTCTTCGTG
**MMP9**	GCCACTACTGTGCCTTTGAGTC	CCCTCAGAGAATCGCCAGTACT

^a^ Abbreviations: VEGFA, vascular endothelial growth factor A; TACR1, tachykinin receptor 1; TAC1, tachykinin precursor 1; CXCL5, C-X-C motif chemokine 5; GAPDH, glyceraldehyde-3-phosphate dehydrogenase; PAD4, peptidyl arginine deiminase 4; MMP9, matrix metallopeptidase 9; NE, neutrophil elastase; MPO, myeloperoxidase.

### 3.5. IL-6 Concentrations

Serum IL-6 concentrations were determined using a commercial enzyme-linked immunosorbent assay (ELISA) kit (IL E-3200, LDN Labor Diagnostika Nord, Germany). All assays were performed according to the manufacturer's protocol, and concentrations were calculated based on the provided standard curve.

To minimize selection bias, we aimed to include all patients with EM from the TLGS who had available matched samples from both study periods. Measurement bias was mitigated by using standardized, validated laboratory protocols, including quantitative PCR (qPCR) and ELISA, for all assays. All laboratory personnel were blinded to the sample phase (pre- or post-pandemic) during RNA extraction, qPCR, ELISA, and neutrophil stimulation experiments. For both qPCR and ELISA, paired pre- and post-pandemic samples from the same individual were always assayed simultaneously in the same analytical batch. No formal batch-effect correction algorithm was applied; paired simultaneous assaying was used as the primary strategy to reduce systematic batch effects.

All blood samples were processed within 2 hours of collection, and serum aliquots were stored at -80°C. Phase 6 samples (2016 - 2018) had a longer storage duration than Phase 7 samples (2021 - 2023), but no additional freeze-thaw cycles were applied beyond the initial aliquoting.

### 3.6. Ethical Approval

All TLGS participants provided written informed consent at enrollment for the collection, biobanking, and future research use of their samples, including this secondary analysis. Ethical approval was obtained from the Ethics Committee of Shahid Beheshti University of Medical Sciences (code: IR.SBMU.ENDOCRINE.REC.1403.015). All procedures were performed in accordance with the committee's guidelines.

### 3.7. Statistical Analysis

Normality of the data distribution was assessed using the Shapiro-Wilk test. Given the non-normal distribution of gene-expression data, group comparisons were performed using the Wilcoxon test. Continuous variables are presented as the median and interquartile range (IQR). No adjustment for potential confounders, such as age, BMI, or batch effects, was performed in the primary analysis because the limited sample size precluded multivariable modeling, and formal sensitivity analyses were not feasible given the non-normal distributions and small number of pairs. The likely direction of residual confounding is uncertain.

Because of the small sample size, no formal power calculation was performed. To provide a precision context, we estimated that with 13 pairs, a Wilcoxon signed-rank test (2-sided α = 0.05) achieves 80% power to detect a standardized effect size, defined as the median of paired differences divided by their standard deviation, of approximately 1.1 or larger, assuming a moderate-to-strong correlation between paired observations. This corresponds to a fold change of approximately 2.0 or greater in genes with typical pre-pandemic interindividual variability observed in our data. Observed fold changes considerably smaller than this, such as those for TAC1 (1.12), VEGFA (1.03), and MPO (1.02), should therefore be interpreted with caution.

Because of the exploratory nature of the study, no formal correction for multiple testing, such as Bonferroni correction, was applied; all P values are descriptive and hypothesis-generating.

Data were analyzed for completeness, and no missing values were present for the laboratory variables measured in this subset. All analyses were conducted using GraphPad Prism version 10 and SPSS version 20, with a 2-sided P value < 0.05 considered statistically significant.

This study was reported in accordance with the Strengthening the Reporting of Observational Studies in Epidemiology (STROBE) guidelines for cohort studies (15) and its explanation and elaboration document (16).

## 4. Results

### 4.1. Demographic Characteristics

The baseline characteristics of the 13 matched women are presented in Table 1. The mean age increased by approximately 3.3 years, from 43.4 to 46.7 years, between phases, whereas BMI remained essentially unchanged (mean difference, +0.01 kg/m^2^). Because each participant served as her own control, formal paired significance tests for these variables were not performed.

### 4.2. Expression Levels of SP, NK1R, VEGF, and CXCL5

To examine changes in the expression of TAC1 (SP), TACR1 (NK1R), VEGF, and CXCL5 after the COVID-19 pandemic, 13 samples from TLGS Phase 6 (pre-pandemic) and Phase 7 (post-pandemic) were analyzed using real-time PCR (Figure 1). No statistically significant differences were detected for any of these genes; however, the small sample size does not exclude true differences (all P > 0.05). Specifically, TAC1 showed a fold change of 1.12 (95% CI, 0.81 - 1.55; P = 0.375), TACR1 showed a fold change of 1.02 (95% CI, 0.75 - 1.39; P = 0.786), VEGFA showed a fold change of 1.03 (95% CI, 0.72 - 1.48; P = 0.414), and CXCL5 showed a fold change of 0.74 (95% CI, 0.45 - 1.20; P = 0.635). These findings are inconclusive.

**Figure 1.**
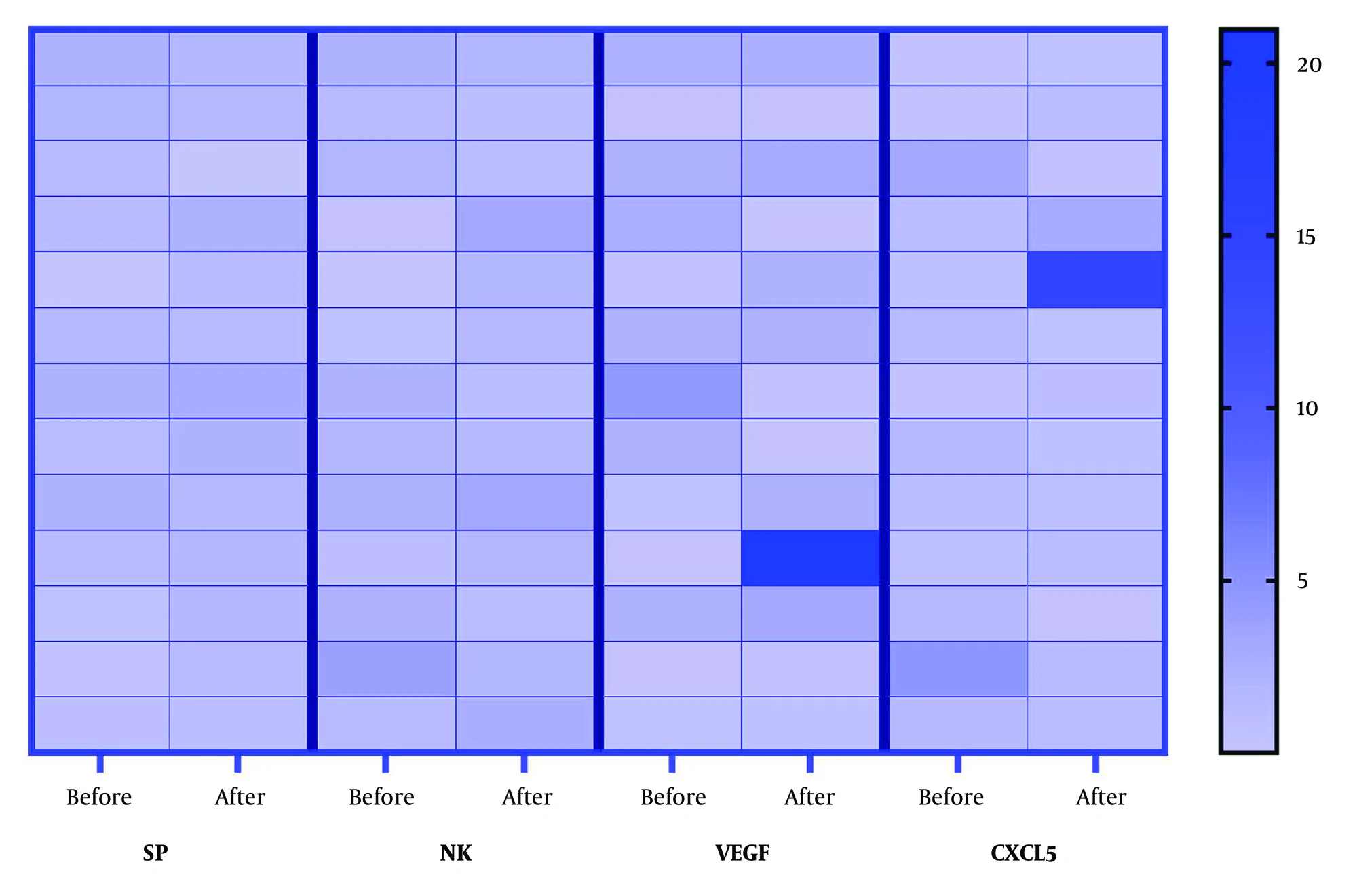
*Relative expression* of TAC1, TACR1, VEGFA, and CXCL5 genes in peripheral blood samples collected before (Phase 6, pre-pandemic) and after (Phase 7, post-pandemic) the COVID-19 pandemic. Gene expression was quantified by real-time PCR; values are normalized relative expression values (2^-ΔCt^, scaled per gene). No statistically significant differences were observed: TAC1 (fold change = 1.12; 95% CI, 0.81 - 1.55; P = 0.375), TACR1 (fold change = 1.02; 95% CI, 0.75 - 1.39; P = 0.786), VEGFA (fold change = 1.03; 95% CI, 0.72 - 1.48; P = 0.414), and CXCL5 (fold change = 0.74; 95% CI, 0.45 - 1.20; P = 0.635). Because of the small sample size, these results do not rule out true differences. Each row represents an individual sample; paired samples are not linked in this display.

### 4.3. NETosis-Related Gene Expression

To assess NETosis induction by serum from patients with EM after the COVID-19 pandemic, serum samples from TLGS Phases 6 and 7 were used to stimulate neutrophils. The expression of NETosis-related genes, including PAD4, MMP9, MPO, and NE, was then evaluated (Figure 2). No significant differences were observed for PAD4 (fold change = 1.23; 95% CI, 0.76 - 2.00; P = 0.921), MMP9 (fold change = 1.40; 95% CI, 0.93 - 2.12; P = 0.084), or MPO (fold change = 1.02; 95% CI, 0.68 - 1.53; P = 0.695). The study had low power to detect fold changes < 2.0; therefore, these non-significant results are inconclusive. In contrast, NE expression was significantly elevated in neutrophils stimulated with post-pandemic serum (fold change = 1.50; 95% CI, 1.01 - 2.25; P = 0.048). This borderline significance should be interpreted cautiously given the small sample size and the multiplicity of tests.

**Figure 2.**
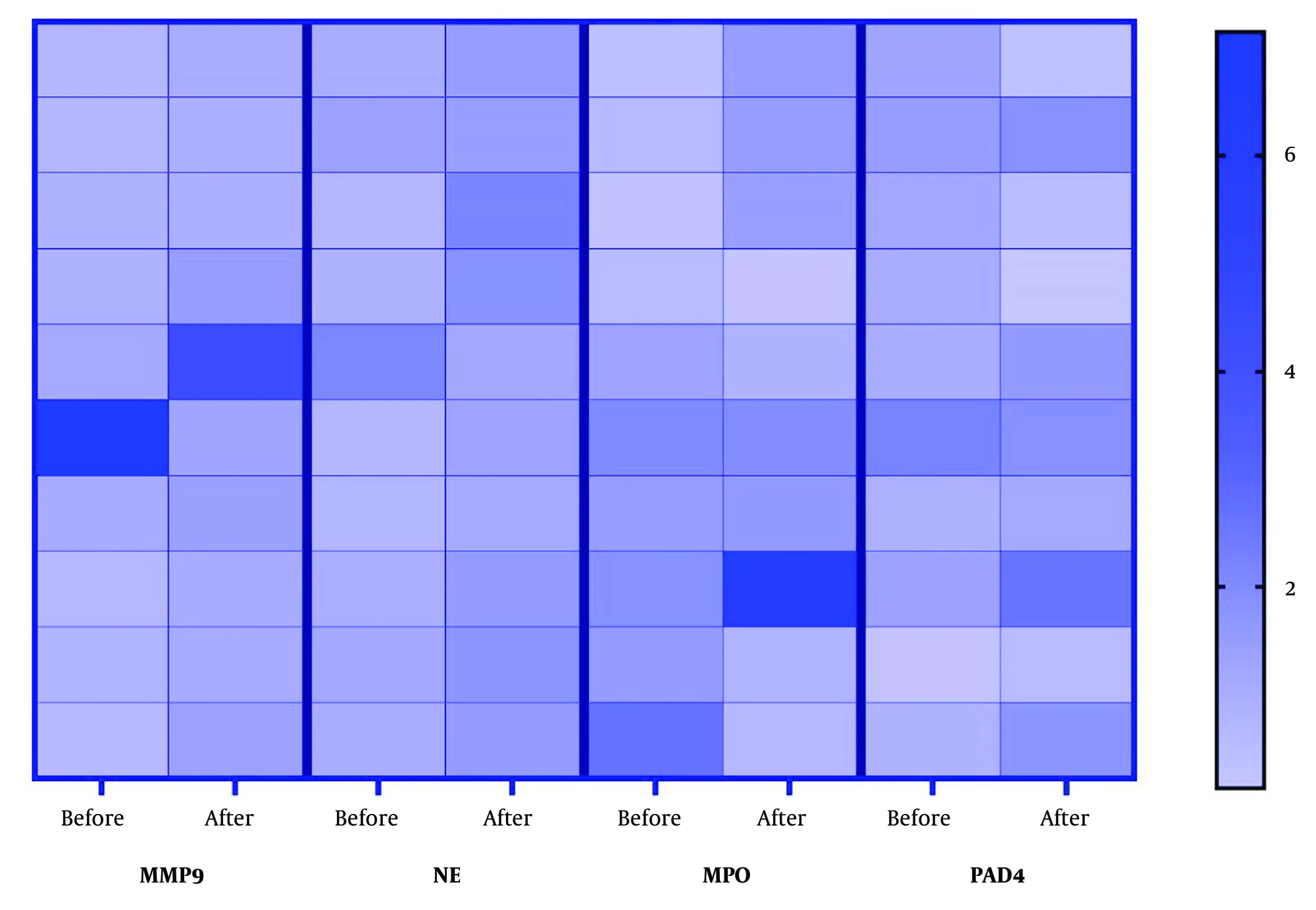
Expression of NETosis-related genes in neutrophils stimulated with sera from individuals with endometriosis before (Phase 6, pre-pandemic) and after (Phase 7, post-pandemic) the COVID-19 pandemic. Values are normalized relative expression values (2^-ΔCt^, scaled per gene). PAD4 (fold change = 1.23; 95% CI, 0.76 - 2.00; P = 0.921), MMP9 (fold change = 1.40; 95% CI, 0.93 - 2.12; P = 0.084), and MPO (fold change = 1.02; 95% CI, 0.68 - 1.53; P = 0.695) did not differ significantly; however, the study had low power for changes < 2.0. NE was significantly elevated (fold change = 1.50; 95% CI, 1.01 - 2.25; P = 0.048), but borderline significance warrants caution. Each row represents an individual sample; paired samples are not linked.

### 4.4. Serum IL-6 Levels

Serum IL-6 levels were numerically higher in Phase 7 than in Phase 6, but the difference was not statistically significant (median paired increase, +0.58 pg/mL; 95% CI for the median paired difference, -0.89 to +2.34 pg/mL; P = 0.750). The wide confidence interval does not exclude a clinically meaningful change; therefore, this finding is inconclusive (Figure 3).

**Figure 3.**
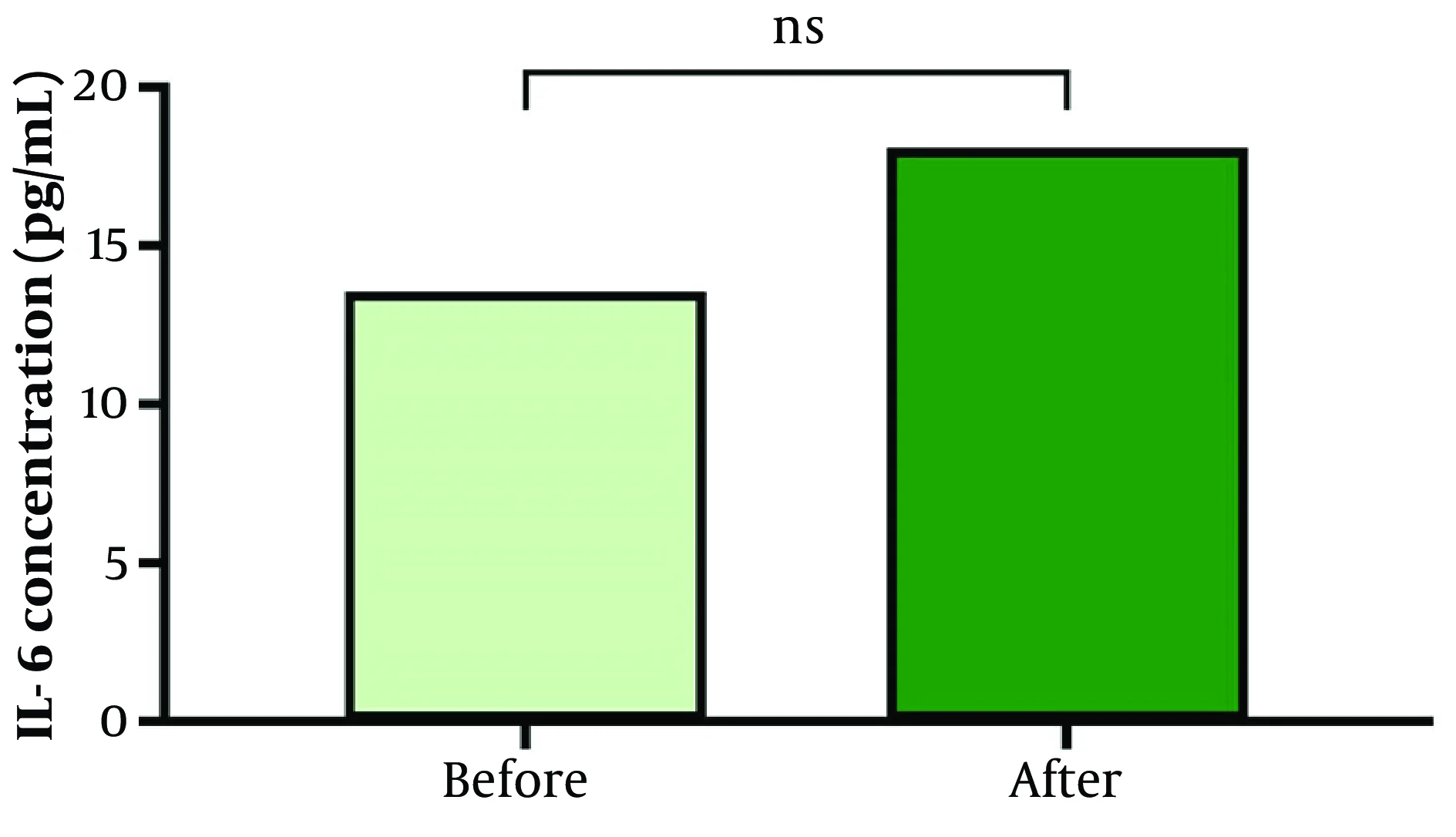
IL-6 concentrations (pg/mL) in serum samples collected before and after the COVID-19 pandemic. The median paired increase was +0.58 pg/mL (95% CI, -0.89 to +2.34 pg/mL; P = 0.750). The difference was not statistically significant, but the confidence interval does not rule out a meaningful change. ns, not significant.

## 5. Discussion

This study examined inflammatory markers in women with EM before and after the COVID-19 pandemic to assess the impact of the pandemic period on the inflammatory profile. Comparison of the pre- and post-pandemic periods revealed a selective increase in neutrophil elastase expression, whereas results for other NETosis markers and IL-6 remained inconclusive. The observed change in NE may reflect composite exposures during the pandemic period, including possible undiagnosed or mild COVID-19 infections, chronic stress, or lifestyle alterations, rather than confirmed infection alone.

The COVID-19 pandemic substantially affected women with EM through multiple pathways involving psychological stress and lifestyle changes. Studies have shown that pandemic-related stress led to worsening EM symptoms, with patients reporting increased pelvic pain, heavy menstrual bleeding, and dysmenorrhea (12). Women experienced high levels of peritraumatic stress and substantial lifestyle modifications, including decreased physical activity and disturbed sleep patterns. The underlying mechanism involves activation of the sympathetic nervous system by psychological stress and increased cortisol levels, which contribute to heightened inflammation and pain sensitivity (17). In addition, anxiety and depression, which became more prevalent during the pandemic, can lower pain tolerance and intensify patients’ subjective experience of symptom severity (18). These findings underscore the importance of a multidimensional approach to EM management, including psychological interventions and lifestyle modifications alongside pharmacological and surgical treatments.

The most notable finding was increased expression of NETosis-related genes in neutrophils stimulated with post-pandemic patient serum, with NE showing a significant increase (P = 0.048). These findings indicate a shift toward heightened neutrophil activity in the post-pandemic period. These changes could be attributed to pandemic-related environmental factors, such as chronic psychosocial stress. Chronic stress alters neutrophil function and, through the release of glucocorticoids, enhances NET formation (19). Moreover, chronic stress can disrupt anti-inflammatory signaling and reduce the ability of glucocorticoids to suppress pro-inflammatory cytokine production. In contrast, other factors, including NK1R, VEGF, and CXCL5, showed no significant differences, suggesting a selective impact on neutrophil-associated pathways.

However, the observed increase in NE expression may also be partially confounded by age, as participants were, on average, 3.3 years older in Phase 7; age-related immune changes can influence neutrophil activity. Without adjusted analyses, we cannot separate pandemic-era effects from aging. Therefore, the observed neutrophil activation could stem from multiple pandemic-related influences rather than confirmed infection alone. Patients with EM, because of their underlying chronic inflammation, may be particularly vulnerable to pandemic-related stimuli, whether viral or non-viral, that activate neutrophils. This could have implications for the disease course and management. Because multiple biomarkers were evaluated and only NE reached borderline significance (P = 0.048), this finding may represent a chance finding due to multiple comparisons and should be interpreted as exploratory until replicated in larger, independent cohorts.

Although serum IL-6 levels were numerically higher after the pandemic, this increase was not statistically significant (P = 0.750), and the wide confidence interval does not exclude a clinically meaningful difference. This finding is inconclusive. As a pivotal pro-inflammatory cytokine, IL-6 is known to play a central role in the pathogenesis of EM by promoting angiogenesis, pain signaling, and lesion survival. Future studies with larger sample sizes are needed to clarify the role of IL-6.

In summary, our data provide preliminary, hypothesis-generating evidence that the post-pandemic period may be associated with increased neutrophil elastase expression in women with EM. Confirmation in larger, well-characterized cohorts is required.

### 5.1. Limitations and Conclusions

This study has several limitations. The small sample size of 13 matched pairs limits the generalizability of the results and the statistical power to detect differences smaller than approximately 2-fold. Consequently, all non-significant comparisons should be considered inconclusive rather than evidence of no change. The borderline significance of NE (P = 0.048) should be interpreted with caution given the multiplicity of tests performed and the small sample size. Although measuring NETosis-related genes in blood provides evidence of activation, it does not directly demonstrate NET presence in endometriotic lesions. Therefore, future immunohistochemistry or immunofluorescence studies on patient tissue samples are recommended to quantify NETosis using specific markers, such as citrullinated histone H3 (CitH3). Future studies should also confirm increased inflammatory marker gene expression in the study groups using other tests, such as ELISA.

It remains challenging to distinguish direct consequences of COVID-19 infection from indirect pandemic-related effects, such as stress and lifestyle changes, on the observed increase in NETosis. Detailed individual-level data on COVID-19 infection history, vaccination status, and pandemic-related stress exposure were not available; therefore, exposure reflects only the post-pandemic time period and cannot be ascribed solely to infection with the virus. The findings should be interpreted in the context of these limitations. Future larger, multicenter studies in diverse populations are needed to confirm these preliminary observations.

This exploratory study identified a preliminary signal of increased neutrophil elastase expression in women with EM during the post-pandemic period, whereas other inflammatory and NETosis markers remained inconclusive. The relative contributions of COVID-19 infection, vaccination, psychosocial stress, and aging cannot be disentangled in this small investigation. These hypothesis-generating findings underscore the need for larger, well-characterized studies that collect individual-level exposure data and account for potential confounders.

## Data Availability

The dataset presented in the study is available on request from the corresponding author during submission or after publication.

## References

[1] Ahn SH, Monsanto SP, Miller C, Singh SS, Thomas R, Tayade C (2015). Pathophysiology and Immune Dysfunction in Endometriosis. Biomed Res Int.

[2] Velho RV, Taube E, Sehouli J, Mechsner S (2021). Neurogenic inflammation in the context of endometriosis-what do we know? International journal of molecular sciences. International Journal of Molecular Sciences.

[3] Hey-Cunningham AJ, Peters KM, Zevallos HB, Berbic M, Markham R, Fraser IS (2013). Angiogenesis, lymphangiogenesis and neurogenesis in endometriosis. Front Biosci (Elite Ed).

[4] Machairiotis N, Vasilakaki S, Thomakos N (2021). Inflammatory Mediators and Pain in Endometriosis: A Systematic Review. Biomedicines.

[5] Yan D, Liu X, Guo SW (2019). Neuropeptides Substance P and Calcitonin Gene Related Peptide Accelerate the Development and Fibrogenesis of Endometriosis. Sci Rep.

[6] Velho RV, Sehouli J, Mechsner S (2023). Mechanisms of peripheral sensitization in endometriosis patients with peritoneal lesions and acyclical pain. Arch Gynecol Obstet.

[7] Berkes E, Oehmke F, Tinneberg HR, Preissner KT, Saffarzadeh M (2014). Association of neutrophil extracellular traps with endometriosis-related chronic inflammation. Eur J Obstet Gynecol Reprod Biol.

[8] Nothnick W, Alali Z (2016). Recent advances in the understanding of endometriosis: the role of inflammatory mediators in disease pathogenesis and treatment. F1000Res.

[9] Sahoo OS, Pethusamy K, Nayek A, Minocha R, Dhar R, Karmakar S (2024). Paradigm of immune dysregulation in coronavirus disease-2019 infection. Exploration of Immunology.

[10] Monsalve DM, Acosta-Ampudia Y, Acosta NG, Celis-Andrade M, Şahin A, Yilmaz AM (2025). NETosis: A key player in autoimmunity, COVID-19, and long COVID. J Transl Autoimmun.

[11] Sharma V, Aden D, Zaheer S, Ranga S (2023). The role of the formation of neutrophil extracellular traps (NETosis) in the pathophysiology and the complications of COVID-19: A review of the literature. Saudi Journal for Health Sciences.

[12] Kabani Z, Ramos-Nino ME, Ramdass PVAK (2022). Endometriosis and COVID-19: A Systematic Review and Meta-Analysis. Int J Mol Sci.

[13] Nicolás I, Martínez-Zamora MÁ, Gracia M, Feixas G, Rius M, Carmona F (2022). Impact of SARS-COV2 Pandemic on Patients with Endometriosis and Their Health Care. J Womens Health (Larchmt).

[14] Hoppenbrouwers T, Autar ASA, Sultan AR, Abraham TE, van Cappellen WA, Houtsmuller AB (2017). in vitro induction of NETosis: Comprehensive live imaging comparison and systematic review. PLOS ONE.

[15] von Elm E, Altman DG, Egger M, Pocock SJ, Gøtzsche PC, Vandenbroucke JP (2008). The Strengthening the Reporting of Observational Studies in Epidemiology (STROBE) statement: guidelines for reporting observational studies. J Clin Epidemiol.

[16] Vandenbroucke JP, von Elm E, Altman DG, Gøtzsche PC, Mulrow CD, Pocock SJ (2007). Strengthening the Reporting of Observational Studies in Epidemiology (STROBE): explanation and elaboration. PLoS Med.

[17] Miller GE, Cohen S, Ritchey AK (2002). Chronic psychological stress and the regulation of pro-inflammatory cytokines: a glucocorticoid-resistance model. Health Psychol.

[18] Black PH (2002). Stress and the inflammatory response: a review of neurogenic inflammation. Brain Behav Immun.

[19] He XY, Gao Y, Ng D, Michalopoulou E, George S, Adrover JM (2024). Chronic stress increases metastasis via neutrophil-mediated changes to the microenvironment. Cancer cell.

